# Comparison of ventriculoperitoneal shunt to lumboperitoneal shunt in the treatment of idiopathic: A monocentric, assessor-blinded, randomized controlled trial

**DOI:** 10.1097/MD.0000000000026691

**Published:** 2021-08-06

**Authors:** Wenyao Cui, Tong Sun, Ke Wu, Chao You, Junwen Guan

**Affiliations:** a Department of Neurosurgery, West China Hospital, Sichuan University, Chengdu, Sichuan, PR China; b Department of Neurosurgery, Xichang Peoples’ Hospital, Liangshan, Sichuan, PR China.

**Keywords:** cerebrospinal fluid shunt, clinical outcomes, efficacy, normal pressure hydrocephalus, safety

## Abstract

**Background:**

Ventriculoperitoneal shunt (VPS) and lumboperitoneal shunt (LPS) remain the mainstay of idiopathic normal pressure hydrocephalus (INPH). There are no randomized controlled trials completed to compare the efficacy of these 2 shunt techniques.

**Methods/design:**

We will conduct a monocentric, assessor-blinded, and randomized controlled trial titled “Comparison of Ventriculoperitoneal Shunt to Lumboperitoneal Shunt for the treatment of Idiopathic Normal Pressure Hydrocephalus: Phase I (COVLINPH-1)” trial and recruit patients at West China Hospital of Sichuan University since June 2021. And this trial is expected to end in December 2030. Eligible participants will be randomly assigned into LPS group and VPS group at ratio of 1:1 followed by evaluation before surgery, 1 month, 12 months, and 5 years after surgery. The primary outcome is the rate of shunt failure within 5 years. The secondary outcomes include modified Rankin Scale (mRS), INPH grading scale (INPHGS), mini-mental state examination (MMSE), and Evans index. We will calculate the rate of favorable outcome, which is defined as shunt success and an improvement of more than 1 point in the mRS at evaluation point. We will also analyze the complications throughout the study within 5 years after shunt insertion.

**Discussion:**

The results of this trial will provide state-of-the-art evidence on the treatment option for patients with INPH, and will also generate the discussion regarding this subject.

**Trial registration number:**

ChiCTR2000031555; Pre-results.

## 1 Introduction

Idiopathic normal pressure hydrocephalus (INPH) is a common geriatric disorder that has unknown causes featured as the triad of Hakim-Adams syndrome (gait/balance disturbance, dementia, and urinary incontinence), accompanying with ventriculomegaly but normal intracranial pressure.^[1,2]^ Early evidence indicated around 1.5% of elderly (>60 years) per year was estimated to develop INPH.^[3,4]^ Although the pathophysiological mechanisms of INPH have not been fully understood, there is a few evidences showing increased arterial pulse pressure might involve in the development of INPH.^[5,6]^

Currently, cerebrospinal fluid (CSF) shunts including ventriculoperitoneal shunt (VPS) and lumboperitoneal shunt (LPS) remain the mainstay of INPH.^[7]^ VPS is the most commonly used and widely studied treatment and LPS is an important and alternative option. Bur growing number of studies recently suggest LPS is the preferred and superior option since several advantages, particularly the avoidance of brain injury. In this regard, LPS has become the first-line and most commonly used methods to treat INPH in Japan.^[8,9]^

Over the past few decades, a great deal of attention has been given with the respect to the best treatment for INPH. There are some studies suggesting no significant differences among the different shunts used, which are mainly retrospective design.^[10]^ Miyajima et al^[11]^ conducted a prospective and multi-center trial on the efficacy and safety of LPS for the treatment of INPH, indicating the incidence of shunt revisions of LPS (7%) is slightly higher compared with a historical trial that test the efficacy of VPS for the treatment of INPH (1%), and despite the risk of shunt failure, LPS could be the superior option considering about the minimally invasiveness. To date, there are no randomized controlled trials completed to compare the efficacy of these 2 shunt techniques, therefore, it is difficult to draw strong conclusions regarding the optimal treatment.^[12]^

## 2 Objective

The objective of this trial is to compare the efficacy and safety of VPS to LPS for the treatment of patients with INPH.

## 3 Methods and design

### 3.1 Ethic and dissemination

This trial will follow the Guidelines for Good Clinical Practice and the Declaration of Helsinki (2002) of the World Medical Association, and implement the principles and requirements of informed consent, privacy protection, research free and compensation, risk control, protection of special subjects and compensation for research related damages. Before the start, the Institutional Review Board of West China Hospital has approved the study on February 20, 2020. The study has registered through Chinese Clinical Trial Registry in April 2020 (ChiCTR2000031555).

Before recruitment, the researcher has the responsibility to completely and comprehensively introduce the purpose, procedure and possible risks of this study to the subject or / and his legal representative, and sign a written informed consent. The subjects should be fully informed that they are completely voluntary to participate in the clinical study, and they can refuse to participate in or withdraw from this study at any stage of the trial. When it comes to discrimination and revenge, their medical treatment and rights will not be affected. Informed consent should be kept as clinical research documents for future reference to protect the privacy and data confidentiality of subjects.

We will share the data on ResMan Research Manager within 6 months after the trial complete and the results will be published in peer-reviewed journals, together with conferences.

### 3.2 Study design

We will conduct a monocentric, assessor-blinded, and randomized controlled trial titled “Comparison of Ventriculoperitoneal Shunt to Lumboperitoneal Shunt for the treatment of Idiopathic Normal Pressure Hydrocephalus: Phase I (COVLINPH-1)” trial and recruit patients at West China Hospital of Sichuan University since June 2021. And this trial is expected to end in December 2030. Eligible participants will be randomly assigned into LPS group and VPS group at ratio of 1:1 followed by evaluation before surgery, 1 month, 12 months, and 5 years after surgery. The primary outcome is the rate of shunt failure within 5 years. The secondary outcomes include modified Rankin Scale (mRS), INPH grading scale (INPHGS), mini-mental state examination (MMSE), and Evans index. We will calculate the rate of favorable outcome, which is defined as shunt success and an improvement of more than 1 point in the mRS at evaluation point. We will also analyze the complications throughout the study within 5 years after shunt insertion.

### 3.3 Recruitment and eligibility

The flow chart of the selection of patients is shown in Fig. 2. Once the eligible participants are admitted, 3-dimension brain and spine magnetic resonance imaging scan will be first performed to further evaluate the ventricles, aqueduct, basal cisterns, and spinal subarachnoid space before shunt surgery. Each participant will receive financial compensation. The inclusion and exclusion criteria are shown as follows:

### 3.4 Inclusion criteria

1. Age >40 years;
2. Idiopathic or insidious onset;
3. At least 2 following symptoms: gait/balance disturbance, impairment in cognition, urinary incontinence.
4. Evans index >0.3;
5. The CSF opening pressure is 70 to 200 mmH_2_O.

### 3.5 Exclusion criteria

1. Secondary hydrocephalus;
2. Obstructive hydrocephalus;
3. Chiari malformation;
4. Parkinson's disease;
5. Alzheimer's disease.

### 3.6 Sample size

A recent meta-analysis systematically reviewed the shunting outcomes in patients with INPH and showed the rate of VPS failure and LPS failure were 18.0% and 14.0%, respectively.^[13]^ Therefore, a sample of 250 for each group will be required in this trial with a significance level of 5% (two-sided) and a power of 80% to demonstrate a 20% difference. Finally, the sample size will be enlarged to 300 for each group.

### 3.7 Randomizing and blinding

This trial is randomized and open-label. We will complete the randomization through a random number table, but the data collectors, accessors, and analysts are blinded to allocation.

### 3.8 Intervention

We will propose the standard procedures for shunt implantation and surgeons will be trained before recruitment. We will utilize the shunt system with programmable pressure valve, which will be set to its highest level before surgery.^[14]^ After shunt insertion, the pressure setting will be lowered by 1-level according to the symptoms or radiological signs.^[15]^

### 3.9 Ventriculoperitoneal shunt

VPS will be performed under general anesthesia and the proximal shunt tubing will be inserted into the right lateral ventricles. A ventricular catheter is inserted into the lateral ventricle. The valve will be placed at the cranial incision with a 3-point fixation to the subcutaneous tissue. A subcutaneous tunneler is made to connect the valve with abdominal cavity. The peritoneal catheter will be inserted if the CSF flow through shunt catheter is observed.

### 3.10 Lumboperitoneal shun

The patients in the left lateral position under general anesthesia. A lumbar catheter is inserted through the L3/4 interlaminar space into the spinal subarachnoid space. A subcutaneous flank region is then made to fix the valve. A subcutaneous tunneler is made to connect the spinal subarachnoid space, frank region, and abdominal cavity. The peritoneal catheter will be inserted if the CSF flow through shunt catheter is observed.

### 3.11 Outcomes

**Figure 1:**
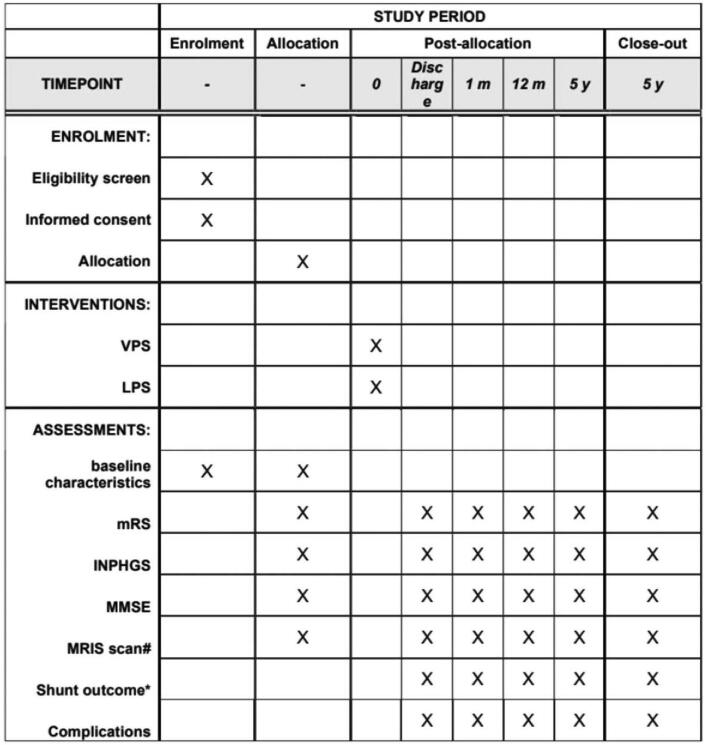
The schedule of enrolment, interventions, and assessments. # The position of MRI scan include brain, lumbar, and abdomen. ∗ “Shunt outcome” includes shunt failure and shunt success. INPHGS = idiopathic normal pressure hydrocephalus grading scale, LPS = lumboperitoneal shunt, MMSE = Mini-Mental State Examination, MRI = magnetic resonance imaging, mRS = modified Rankin Scale, VPS = ventriculoperitoneal shunt.

Evaluation schedule is shown in Fig. 1. Each participant is evaluated before surgery, 1 month, 12 months, and 5 years after surgery by 2 independent assessors.

**Figure 2:**
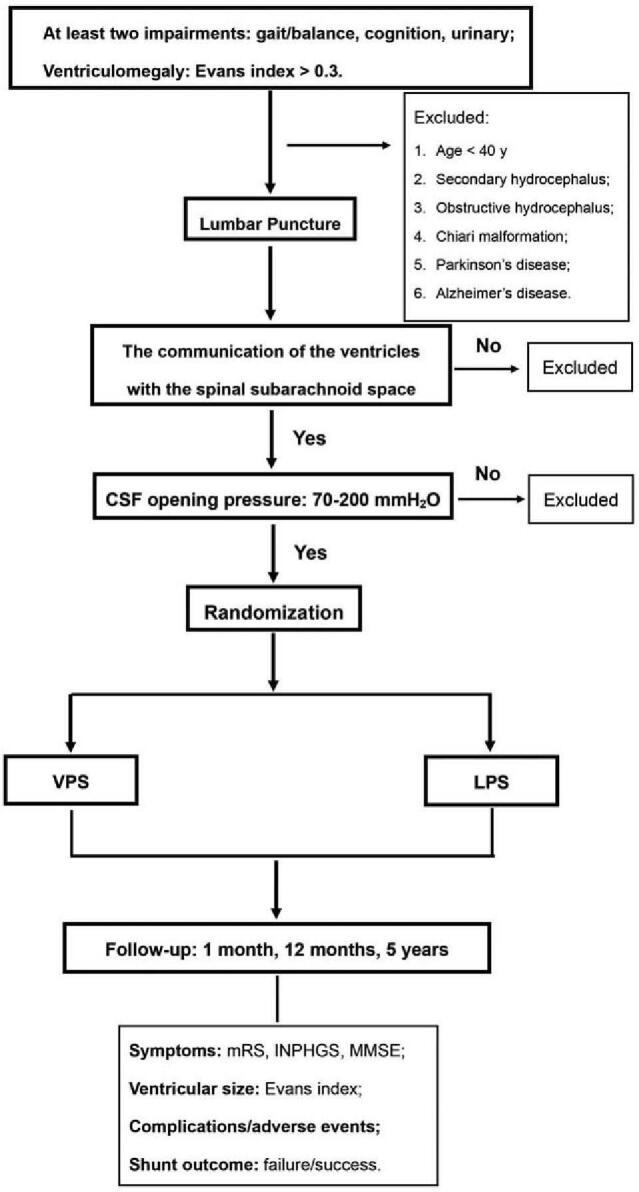
The flow chart of the selection pf patients. CSF = cerebrospinal fluid, INPHGS = idiopathic normal pressure hydrocephalus grading scale, LPS = lumboperitoneal shunt, MMSE = Mini-Mental State Examination, mRS = modified Rankin Scale, VPS = ventriculoperitoneal shunt.

### 3.12 Primary outcome

The primary outcome is the rate of shunt failure within 5 years. According to previous studies, shunt failure is defined as the presence of shunt obstruction, breakage, exposure, misconnection, infection, or any conditions requiring shunt revision. Shunt success is defined as improvement without shunt revision.^[16]^

### 3.13 Secondary outcomes

The secondary outcomes include mRS, INPHGS, MMSE, and Evans index at 1 month, 12 months, and 5 years after surgery. We will calculate the rate of favorable outcome, which is defined as shunt success and an improvement of more than 1 point in the mRS at evaluation point. Evans index is tested thorough magnetic resonance imaging scan. We will also analyze the complications throughout the study within 5 years after shunt insertion.

Safety indicators include any serious adverse event, which is defined as death, consciousness disorders, admission to intensive care unit, deterioration, or an extension of the length of stay, or disability, which is associated with shunt surgery.^[17]^

### 3.14 Data collection and management

Two independent data collectors will collect the baseline information (age, sex, comorbidities, recruitment time, CSF opening pressure, clinical manifestations, radiological features) at the time of admission, perioperative conditions, and follow-up outcomes at each evaluation point (shunting outcomes, mRS, INPHGS, MMSE, Evans index, complications, serious adverse events). The data is first recorded in paper and then stored in Excel.

### 3.15 Statistics analysis

We will use SPSS 19.0 software to analyze the data. The statistical description of categorical variables adopts number (percentage). For continuous data, firstly, the Kolmogorov Smirnov test will be used to analyze the normality. The statistical description in accordance with the normal distribution is mean ± standard deviation (SD), and the median (range) description is used for those do not follow the normal distribution. Wilcoxon rank sum test or *t* test is used to compare the difference of continuous data according to the normality. Chi-Squared test is used to compare the difference of categorical variables. Kaplan–Meier survival analysis is used to draw the shunt-success curve. In this study, *P* value under .05 is considered to be statistically significant.

### 3.16 Data monitoring

An independent data monitoring committee (DMC) consisting of neurologists, assessors, and data analysts from West China Hospital will check the data once a year to guarantee the efficacy and safety and of this trial.

All adverse events will be recorded in detail, properly handled and tracked until they were properly resolved or stable, and be timely reported to ethics committee and competent department. We will conduct cumulative review on all adverse events on a regular basis, and convene a meeting of researchers to assess the risk of the study if necessary.

## 4 Discussion

This study is designed to highlight the controversy concerning INPH treatment options. To address the highlight, each participant will be followed up to 5 years after shunts to compare the long-term outcomes of VPS cohorts with that of LPS cohorts. The improvement of symptoms, brain imaging, the rate of shunt failure, complications, together along with the adverse events throughout the study would be investigated. The study could provide evidence for the selection of treatments for patients with INPH and may generate discussion about the optimal treatment. Despite of the strengths, there are still some questions that need to be discussed.

The diagnosis of INPH remains controversial since the lack of widely accepted and standardized criteria. Marmarou et al first proposed the evidence-based guidelines for clinical diagnosis of INPH in 2005 (Western guideline).^[18]^ Japanese Society of Normal Pressure Hydrocephalus published the English edition of the Guidelines for Management of Idiopathic Normal Pressure Hydrocephalus in 2008 (Japanese guideline), which was then updated in 2012.^[19]^ Despite of the fact that there are some differences between Western guideline and Japanese guideline, it is rather widely accepted by many clinicians that the diagnosis of INPH should be classified into probable, possible, and unlikely categories based on clinical history, symptoms, and brain imaging, and an accurate diagnosis of INPH depends upon the response to shunting.^[18]^

After the description of the triad of syndrome by Hakim and Adams, the clinical symptoms of INPH are essential regarding its diagnosis, among which gait/balance disturbance tends to be the most readily recognized and common while cognition defects and urinary symptoms do not occur in all patients. As a consequence, either Western guideline or Japanese guideline suggested the probable INPH patients should present at least 2 symptoms of Hakim-Adams syndrome and gait/balance disturbance must be present.

Ventriculomegaly (Evans index >0.3) is another common and obligate feature of INPH. Recently, a great deal of attention is given to the radiological sign of Disproportionately Enlarged Subarachnoid Space Hydrocephalus (DESH sign, high convexity tightness and dilated sylvian fissure with ventriculomegaly) on coronal magnetic resonance imaging, which could be used to distinguish INPH from other neurological disorders, such as Parkinson's disease and Alzheimer's disease.^[19]^

In terms of the definition of “normal pressure,” it is different between Western guideline and Japanese guideline. The CSF opening pressure measured by lumbar puncture in the lateral recumbent position is in the range of 70 to 245 mm H_2_O according to the Western guideline, comparing to a CSF opening pressure under 200 mm H_2_O based on Japanese guideline. Taken together, a range of 70 to 200 mm H_2_O is considered in this study since pressures that are dramatically higher or lower than this range are not accordant with a probable NPH diagnosis, as well as not suitable for the upcoming LPS surgery.

This trial will the first randomized controlled trial that compares the long-term outcomes of VPS with that of LPS in the treatment of INPH. The results of this trial will provide certain evidence for the treatment option for patients with INPH. This trial will also generate the discussion regarding this subject. In addition, based on this trial, we could analyze the related factors of shunt failure in patients with INPH to exclude the patients with high risk of shunt failure.

This trial has some limitations. First, it is single-center study. Second, the shunting outcomes are possibly associated with personal experiences and qualifications. In this regard, we will propose a uniform standard and surgeons will be trained before recruitment.

## Author contributions

**Conceptualization:** Wenyao Cui, Tong Sun, Junwen Guan.

**Data curation:** Wenyao Cui.

**Formal analysis:** Ke Wu.

**Funding acquisition:** Chao You.

**Software:** Ke Wu.

**Supervision:** Chao You, Junwen Guan.

**Writing – original draft:** Wenyao Cui, Tong Sun.

**Writing – review & editing:** Chao You, Junwen Guan.
